# Healthcare resource utilisation and associated costs in patients with eosinophilic granulomatosis with polyangiitis in England: A retrospective observational cohort study^☆^

**DOI:** 10.1016/j.waojou.2026.101457

**Published:** 2026-09-02

**Authors:** Salman H. Siddiqui, Bo Ding, Paul Dolin, Chris Edmonds, Priya Jain, Jennifer Rowell, Lotte Westerink, Alessandra Lacetera, Pablo Suárez-Sánchez, Cono Ariti, Bélène Podmore, Alvaro Kitchin Velarde, Stephanie Y. Chen

**Affiliations:** a National Heart and Lung Institute, NIHR Imperial Biomedical Research Centre, Imperial College London, Du Cane Road, London, W12 0NN, UK; b BioPharmaceuticals Medical, AstraZeneca, Pepparedsleden 1 Mölndal, Gothenburg, 431 83, Sweden; c BioPharmaceuticals Medical, AstraZeneca, 136 Hills Road, Cambridge, CB2 8PA, UK; d Market Access and Pricing, AstraZeneca, 1 Medimmune Way, Gaithersburg, MD 20878, USA; e Health Economics & Payer Evidence, AstraZeneca, 136 Hills Road, Cambridge, CB2 8PA, UK; f OXON Epidemiology, Calle Del Dr. Fleming, 51, Chamartín, 28036, Madrid, Spain; g BioPharmaceuticals Medical, AstraZeneca, 1 Medimmune Way, Gaithersburg, MD 20878, USA

**Keywords:** Eosinophilic granulomatosis with polyangiitis, Health care costs, Health resources, Health care system

## Abstract

**Background:**

Real-world data on the healthcare resource utilisation (HCRU) and cost burden of eosinophilic granulomatosis with polyangiitis (EGPA) are limited. We assessed all-cause HCRU and costs in patients with EGPA versus a matched general population cohort without EGPA and a severe uncontrolled asthma (SUA) cohort.

**Methods:**

Primary care data in England from the Clinical Practice Research Datalink Aurum database, with linkage to Hospital Episode Statistics inpatient, outpatient and emergency department records, were analysed. Patients with a new EGPA diagnosis in 2006–2020 and ≥ 1 year of data before diagnosis (index date [ID]) were included and matched using a matching ratio of up to 1:4 with a general population cohort without EGPA and patients with SUA. Follow-up was from ID until deregistration, last data collection, death or study end. HCRU and associated costs were assessed across the 12 months prior to ID, and annually from ID to end of the study period, and by disease states and Five-Factor Score [FFS].

**Results:**

A total of 486 patients with EGPA were identified, with a corresponding matched general population cohort of 1938 and SUA cohort of 1005 patients. Annual all-cause HCRU rates during follow-up were higher in the EGPA cohort versus the general population and SUA cohorts for all types of care, particularly in the first year after ID. Patients with an FFS of 0 generally had lower HCRU than those with an FFS ≥1. Annualised total HCRU-associated costs (95% confidence interval [CI]) were higher in the EGPA cohort (£13,978 [12,068, 15,888]) versus the general population (£2303 [2145, 2461]) and matched SUA cohorts (£3571 [3326, 3816]), with cost ratios (95% CI) of 8.3 (7.1, 9.6) and 4.1 (3.6, 4.6) respectively, both p < 0.0001. The greatest cost driver was hospital admissions with cost ratios (95% CI) of 12.3 (9.8, 15.5) and 5.8 (4.7, 7.2) compared with the general population and SUA cohorts, respectively. Median all-cause annualised total costs per patient were 46% and 56% higher among patients with relapse or stable disease, respectively, than among those in remission at ID.

**Conclusion:**

Patients with EGPA incurred significantly higher annualised HCRU rates and associated costs than both the general population and SUA cohorts, underscoring a substantial clinical and economic burden and highlighting a persistent unmet clinical need in practice.

## Introduction

Eosinophilic granulomatosis with polyangiitis (EGPA) is a complex and rare disease characterised by necrotising vasculitis of small-to-medium-sized blood vessels and eosinophilic granulomatous inflammation, which is associated with adult-onset asthma.^1^^,^^2^ The prevalence and incidence of EGPA are wide ranging, estimated in a recent systematic literature review and meta-analysis to be 34.44 per million and 2.15 cases per million person-years (PY), respectively.^3^ In the United Kingdom (UK), a retrospective longitudinal study estimated the prevalence of EGPA to range from 22.7 to 45.6 cases per million people during the period 2005 to 2019; during the years 2006–2019, the incidence was reported to range from 2.3 to 4.0 per million PY.^4^ EGPA is characterised by a relapsing and remitting disease course, with relapse occurring in 25–49% of cases despite initial remission.^1^ Oral glucocorticoids have long been the cornerstone of EGPA treatment, irrespective of prognosis.^1^^,^^5^ However, achieving and maintaining remission often necessitates the addition of other immunosuppressants or novel biological agents. Moreover, the long-term use of oral glucocorticoids and immunosuppressants is commonly associated with a considerable cumulative toxicity burden, including increased risk of infection, osteoporosis, diabetes, cardiovascular disease and impaired health-related quality of life.^6^^,^^7^ Notably, cumulative exposure to oral glucocorticoids contributes substantially to long-term morbidity, with doses exceeding 935 mg linked to an 80% likelihood of clinically meaningful glucocorticoid-related toxicity.^7^ Taken together, the substantial clinical burden associated with EGPA highlights the importance of timely and accurate diagnosis, as well as optimised disease management strategies to improve patient outcomes and reduce long-term complications.

Available evidence highlights the substantial clinical^8^ and economic burden of EGPA.^4^^,^^9^^,^^10^ In a recent retrospective cohort study conducted in the United States (US), over one-third of patients with EGPA experienced relapse.^9^ Compared with patients with asthma alone, those with EGPA had significantly higher healthcare resource utilisation (HCRU) and greater systemic glucocorticoid use, with all-cause healthcare costs reported to be 2.5 times greater.^9^ This increased cost burden was largely driven by a significantly higher proportion of patients with EGPA requiring emergency hospital visits and inpatient admissions, as well as increased pharmacy expenditures compared with those with asthma alone. Another recent US-based analysis comparing HCRU and costs among patients with EGPA, those without EGPA and those with severe uncontrolled asthma (SUA)^11^ revealed similar trends, identifying inpatient admissions as the primary driver of overall costs.

There remains a need for real-world data on HCRU and costs associated with managing EGPA to better understand the overall impact of the disease. In particular, evidence is lacking on how different EGPA disease states influence HCRU and related costs. In previous research using the Clinical Practice Research Datalink (CPRD) Aurum database, the patient journey, including mortality, survival, and transitions between disease states (remission, stable disease, and relapse) was examined among patients in England with EGPA.^12^, ^13^, ^14^ In this study, we quantify HCRU and associated costs in patients with EGPA, comparing outcomes with matched cohorts from the general population and individuals with SUA, as well as across different EGPA disease states.

## Methods

### Study design

This study forms part of the CONSTELLATION real-world evidence programme focused on rare eosinophil-associated disease. This was a retrospective cohort analysis that used data from the CPRD Aurum primary care database, which is linked to the Hospital Episodes Statistics inpatient, outpatient, and accident and emergency (A&E) records and the Office for National Statistics (ONS) mortality data from England. The protocol (22_001934) was approved by the Independent Scientific Advisory Committee. Full details of the study design, including identification of the EGPA cohort, have been previously reported.^15^ In brief, patients with a first diagnosis of EGPA (Systematized Nomenclature of Medicine Clinical Terms [SNOMED CT] 82275008 or International Classification of Diseases [ICD]-10 M30.1) at the "index date" (ID) were included in the incident EGPA cohort between January 1, 2006 to February 28, 2020 (study period). Additionally, 2 matched patient cohorts were randomly selected from the CPRD database: a general population cohort and a SUA cohort. Matching was based on general practitioner (GP) practice, year of birth, and sex using a matching ratio of up to 1:4 (ie, one incident EGPA patient to up to 4 general or SUA patients). The ID assigned to each matched cohort was based on the incident EGPA patient's diagnosis date. Both matched cohorts included patients with no diagnosis of EGPA at any time, who were alive at the matched ID of the patient with incident EGPA, and had ≥1 year of medical records prior to and including the ID; the SUA cohort included patients who had at least 1 asthma diagnosis code AND met the SUA criteria in the CPRD database on or after January 1, 2006 (Supplemental Methods 1).^16^

### Outcomes

#### HCRU and costs

HCRU and costs were calculated and compared between the incident EGPA cohort and the matched general population and SUA cohorts for the whole follow-up period (after the ID and during the study period). All-cause HCRU and costs were quantified for the 3 cohort groups, including in the incident EGPA cohort by disease state (remission, stable disease, and relapse) in the prior 6 months (Supplemental Methods 2), as well as by the 1996 Five-Factor Score (FFS)^17^ (score of 0 or ≥ 1; Supplemental Methods 3).

HCRU study outcomes included outpatient care (general practice consultations, defined as all consultations at a general practice, including those with a GP and those with a nurse; GP prescriptions; and number of outpatient visits), A&E visits, and hospital admissions (number of inpatient admissions; length of hospital and intensive care unit [ICU] stays in days). The costs calculated included the following: general practice consultations (including with a GP or nurse), GP prescription costs, hospital admissions, critical care admissions, outpatient visits, and A&E visits, as well as total annual costs of all HCRU for the previously listed categories. Cost analyses were based on individual-level resource utilisation and were conducted in 2 ways: (1) using all individuals at the beginning of the period as the denominator, and (2) considering only those individuals who utilised the resource at least once (patients with resource use). Costs were computed by multiplying the number of units used by each case by their unit price and then by aggregating (summing) particular resource cost variables. The National schedule of National Health Service costs was used to obtain the national average unit cost of the respective services utilised (Supplemental Methods 4**;** calendar year 2022 and financial year 2022–2023 [UK £]). Prescription unit costs were based on the ‘Prescription Cost Analysis’ and Drug Tariff Part VIII;^18^^,^^19^ if a cost could not be assigned, a manual search in the Dictionary of Medicines and Devices browser or the CPRD Aurum cost variable was used.^20^

HCRU and associated costs were analysed across 2 time periods: the 12 months prior to the ID, and annually from ID to the end of the study period. HCRU was treated as count data and summarised using per-patient cumulative distributions for each resource type, across both periods. HCRU was described by the usage rates of each resource type, calculated using the total time each patient contributed to the analysis period, expressed in patient-years. The proportion of patients utilising each HCRU category was also reported.

#### Statistical analysis

All statistical descriptive summaries and analyses were performed using SAS Version 9.4 (TS1M6) or higher.

All outcomes described were summarised using descriptive techniques through the tabular display of the number of valid cases, means and standard deviations (SDs), medians and interquartile range (IQR; Q1–Q3), minimum and maximum values for continuous variables, and frequency distributions and percentages for categorical and binary variables. HCRU rates were computed as overall yearly rates for the study period and modelled by time using a Poisson regression analysis. When applicable, 95% confidence intervals (CIs) were calculated. When the incident EGPA cohort was compared to the matched general population and SUA cohorts, Chi-square and Fisher's exact tests were used to compare categorical variables. All statistical tests were 2-sided and conducted at the 0.05 alpha level, unless otherwise stated. According to CPRD policy and to protect patient confidentiality, any analysis resulting in fewer than 5 events was not reported and is presented as “<5”.

## Results

### Baseline demographics and clinical characteristics

In total, 486 patients with a first diagnosis of EGPA during the study period were included in the incident EGPA cohort; 1938 matched individuals were included in the general population cohort and 1005 in the SUA cohort (Supplemental Table 1 and Supplemental Fig. 1). Asthma was the most frequent comorbidity in the incident EGPA cohort, affecting 79.8% of patients – higher than the rate observed in the matched general population cohort (10.1%) (Table 1). Bronchiectasis was more prevalent in the incident EGPA cohort (11.9%) compared with both the matched general population (0.4%) and SUA (2.2%) cohorts (p < 0.001 for both comparisons). Similarly, eosinophilic conditions were more frequently reported in the incident EGPA cohort (16.5%) than in the matched general population (5.4%) or SUA cohorts (10.0%) (p < 0.001 for both comparisons). Nasal polyposis also occurred more often in the incident EGPA cohort (18.9%) compared with the matched general population (0.7%) and SUA (3.0%) cohorts (p < 0.001 for both comparisons).

**Table 1:** Comorbidities in the EGPA cohort compared with the matched general population and SUA cohorts.

Comorbidities	Incident EGPA cohort	Matched general population cohort				Matched SUA cohort			
	n (%), N = 486	n (%), N = 1938	Prevalence ratio	95% CI	p-value^a^	n (%), N = 1005	Prevalence ratio	95% CI	p-value^a^
**Patients with ≥1 comorbidity, n (%)**	**472 (97.1)**	**1145 (59.1)**	**1.6**	**1.6**, **1.7**	**<0.001**	**1005 (100.0)**	**1.0**	**1.0**, **1.0**	**<0.001**
**Obstructive airway disease**	**414 (85.2)**	**241 (12.4)**	**6.9**	**6.1**, **7.8**	**<0.001**	**1005 (100.0)**	**0.9**	**0.8**, **0.9**	**<0.001**
Asthma	388 (79.8)	196 (10.1)	7.9	6.9, 9.1	<0.001	1005 (100.0)	0.8	0.8, 0.8	<0.001
Bronchiectasis	58 (11.9)	7 (0.4)	33.0	15.2, 71.9	<0.001	22 (2.2)	5.5	3.4, 8.8	<0.001
Chronic obstructive pulmonary disease	92 (18.9)	77 (4.0)	4.8	3.6, 6.3	<0.001	189 (18.8)	1.0	0.8, 1.3	0.954
Cystic fibrosis with pulmonary manifestations	0 (0.0)	0 (0.0)	–	–	–	<5	–	–	–
**Other eosinophilic conditions**	80 (16.5)	105 (5.4)	3.0	2.3, 4.0	<0.001	100 (10.0)	1.7	1.3, 2.2	<0.001
Atopic dermatitis or eczema	36 (7.4)	105 (5.4)	1.4	0.9, 2.0	0.093	99 (9.9)	0.8	0.5, 1.1	0.127
Diffuse eosinophilic fasciitis	0 (0.0)	0 (0.0)	–	–	–	0 (0.0)	–	–	–
Eosinophilic cellulitis	0 (0.0)	0 (0.0)	–	–	–	0 (0.0)	–	–	–
Eosinophilic colitis	0 (0.0)	0 (0.0)	–	–	–	0 (0.0)	–	–	–
Eosinophilic endomyocardial disease	<5	0 (0.0)	–	–	–	0 (0.0)	–	–	–
Eosinophilic esophagitis	<5	0 (0.0)	–	–	–	0 (0.0)	–	–	–
Eosinophilic gastritis or gastroenteritis	0 (0.0)	0 (0.0)	–	–	–	0 (0.0)	–	–	–
Hypereosinophilic syndrome	<5	0 (0.0)	–	–	–	0 (0.0)	–	–	–
Pulmonary eosinophilia	40 (8.2)	0 (0.0)	–	–	–	<5	–	–	–
**Malignancies and myeloproliferative diseases**	74 (15.2)	194 (10.0)	1.5	1.2, 2.0	<0.001	117 (11.6)	1.3	1.0, 1.7	0.052
**Other comorbidities**	385 (79.2)	978 (50.5)	1.6	1.5, 1.7	<0.001	707 (70.3)	1.1	1.1, 1.2	<0.001
Nasal polyposis	92 (18.9)	13 (0.7)	28.2	15.9, 50.0	<0.001	30 (3.0)	6.3	4.3, 9.4	<0.001
Ischaemic heart disease	59 (12.1)	125 (6.4)	1.9	1.4, 2.5	<0.001	104 (10.3)	1.2	0.9, 1.6	0.298
Arrhythmia	34 (7.0)	91 (4.7)	1.5	1.0, 2.2	0.04	47 (4.7)	1.5	1.0, 2.3	0.065
Deep vein thromboembolism	16 (3.3)	23 (1.2)	2.8	1.5, 5.2	0.002	11 (1.1)	3.0	1.4, 6.4	0.005
Arterial thrombosis	5 (1.0)	5 (0.3)	4.0	1.2, 13.7	0.028	<5	–	–	–
Dyslipidaemia	45 (9.3)	142 (7.3)	1.3	0.9, 1.7	0.152	83 (8.3)	1.1	0.8, 1.6	0.517
Obesity	31 (6.4)	101 (5.2)	1.2	0.8, 1.8	0.31	117 (11.6)	0.5	0.4, 0.8	0.002
Hyperthyroidism	9 (1.9)	10 (0.5)	3.6	1.5, 8.8	0.005	5 (0.5)	3.7	1.3, 11.0	0.018
Hypothyroidism	39 (8.0)	100 (5.2)	1.6	1.1, 2.2	0.015	71 (7.1)	1.1	0.8, 1.7	0.506
Vitamin D deficiency	15 (3.1)	29 (1.5)	2.1	1.1, 3.8	0.021	28 (2.8)	1.1	0.6, 2.1	0.745
Gastro-oesophageal reflux disease	42 (8.6)	62 (3.2)	2.7	1.8, 3.9	<0.001	60 (6.0)	1.4	1.0, 2.1	0.056
Irritable bowel syndrome	12 (2.5)	47 (2.4)	1.0	0.5, 1.9	0.955	29 (2.9)	0.9	0.4, 1.7	0.645
Allergic rhinitis	83 (17.1)	59 (3.0)	5.6	4.1, 7.7	<0.001	103 (10.2)	1.7	1.3, 2.2	<0.001
Interstitial pulmonary disease	24 (4.9)	<5	–	–	–	5 (0.5)	9.9	3.8, 25.9	<0.001
Throat and chest pain	136 (28.0)	312 (16.1)	1.7	1.5, 2.1	<0.001	264 (26.3)	1.1	0.9, 1.3	0.482
Back pain (dorsalgia)	90 (18.5)	287 (14.8)	1.3	1.0, 1.6	0.041	207 (20.6)	0.9	0.7, 1.1	0.349
Anxiety	41 (8.4)	123 (6.3)	1.3	0.9, 1.9	0.1	124 (12.3)	0.7	0.5, 1.0	0.027
Depression	43 (8.8)	159 (8.2)	1.1	0.8, 1.5	0.646	152 (15.1)	0.6	0.4, 0.8	0.001
Insomnia	17 (3.5)	48 (2.5)	1.4	0.8, 2.4	0.214	43 (4.3)	0.8	0.5, 1.4	0.474
Obstructive sleep apnoea	7 (1.4)	11 (0.6)	2.5	1.0, 6.5	0.053	18 (1.8)	0.8	0.3, 1.9	0.622
**Autoimmune disorders**									
Ankylosing spondylitis	<5	<5	–	–	–	<5	–	–	–
Antiphospholipid syndrome	0 (0.0)	<5	–	–	–	0 (0.0)	–	–	–
Biliary cirrhosis	0 (0.0)	<5	–	–	–	<5	–	–	–
Chronic inflammatory demyelinating polyneuropathy	0 (0.0)	0 (0.0)	–	–	–	0 (0.0)	–	–	–
Dermatomyositis	<5	<5	–	–	–	<5	–	–	–
Guillain-Barre syndrome	<5	0 (0.0)	–	–	–	0 (0.0)	–	–	–
Idiopathic thrombocytopenic purpura	5 (1.0)	<5	–	–	–	0 (0.0)	–	–	–
Inflammatory bowel disease (Crohn's disease & ulcerative colitis)	14 (2.9)	17 (0.9)	3.3	1.6, 6.6	<0.001	12 (1.2)	2.4	1.1, 5.2	0.024
Sjogren's syndrome	<5	<5	–	–	–	<5	–	–	–
Multiple sclerosis	0 (0.0)	8 (0.4)	–	–	–	6 (0.6)	–	–	–
Myasthenia gravis	0 (0.0)	0 (0.0)	–	–	–	0 (0.0)	–	–	–
Pemphigoid	0 (0.0)	<5	–	–	–	<5	–	–	–
Pemphigus	0 (0.0)	0 (0.0)	–	–	–	<5	–	–	–
Polymyositis	<5	0 (0.0)	–	–	–	0 (0.0)	–	–	–
Psoriasis or psoriatic arthritis	7 (1.4)	30 (1.5)	0.9	0.4, 2.1	0.863	20 (2.0)	0.7	0.3, 1.7	0.458
Rheumatoid arthritis	22 (4.5)	35 (1.8)	2.5	1.5, 4.2	<0.001	24 (2.4)	1.9	1.1, 3.3	0.027
Sarcoidosis	<5	<5	–	–	–	<5	–	–	–
Systemic lupus erythematosus	<5	<5	–	–	–	0 (0.0)	–	–	–
Systemic sclerosis (scleroderma)	0 (0.0)	0 (0.0)	–	–	–	0 (0.0)	–	–	–

Frequencies <5 were masked to protect patient confidentiality.

CI, confidence interval; EGPA, eosinophilic granulomatosis with polyangiitis; SUA, severe uncontrolled asthma.

a P-value from unadjusted log-binomial model.

### HCRU and associated costs in EGPA

Table 2 summarises HCRU among patients in the incident EGPA cohort during 3 periods: 12 months prior to the ID, 12 months post-ID, and the total post-ID period. Rates of general practice consultations (overall, or with a GP), A&E visits, hospital admissions (duration ≥1 day) and hospital readmissions within 30 days were higher during the 12 months prior to the ID than in the total post-ID period. In contrast, outpatient visits, nurse visits, and GP prescriptions increased in the total period post-ID compared with during the 12 months prior to the ID, while rates for other categories of HCRU remained stable.

**Table 2:** All-cause HCRU in the incident EGPA cohort during the 12 months prior to ID, total post-ID period, and 12 months post-ID, among patients with resource usage.

**Source of HCRU**	12 months prior to ID			Total post-ID period			12 months post-ID		
	**Patients with resource usage, n (%)**	**Rate per PY (all patients)**	**95% CI**	**Patients with resource usage, n (%)**	**Rate per PY (all patients)**	**95% CI**	**Patients with resource usage, n (%)**	**Rate per PY (all patients)**	**95% CI**
**Outpatient care**									
GP consultations^a^	450 (92.6)	11.8	11.5, 12.1	467 (96.1)	9.7	9.5, 9.8	446 (91.8)	12.1	11.8, 12.4
GP	440 (90.5)	10.6	10.3, 10.9	467 (96.1)	8.0	7.9, 8.1	440 (90.5)	10.4	10.1, 10.7
Nurse	159 (32.7)	1.2	1.1, 1.3	301 (61.9)	1.6	1.6, 1.7	171 (35.2)	1.7	1.5, 1.8
GP prescriptions (all drugs of interest^b^)	476 (97.9)	39.2	38.6, 39.7	473 (97.3)	57.1	56.8, 57.3	468 (96.3)	55.3	54.7, 56.0
Systemic glucocorticoids^c^	333 (68.5)	4.0	3.8, 4.2	421 (86.6)	7.5	7.4, 7.7	398 (81.9)	8.2	7.9, 8.5
Oral glucocorticoids	331 (68.1)	4.0	3.8, 4.2	419 (86.2)	7.5	7.4, 7.6	397 (81.7)	8.2	7.9, 8.5
IV/IM glucocorticoids	7 (1.4)	0.0	0.0, 0.0	15 (3.1)	0.0	0.0, 0.0	<5	–	–
Immunosuppressants^d^	45 (9.3)	0.6	0.5, 0.7	261 (53.7)	4.3	4.2, 4.3	210 (43.2)	3.6	3.4, 3.8
Outpatient visits^e^	421 (86.6)	7.5	7.2, 7.7	474 (97.5)	9.2	9.1, 9.4	462 (95.1)	13.7	13.4, 14.1
A&E visits	235 (48.4)	1.1	1.1, 1.2	377 (77.6)	0.7	0.7, 0.7	201 (41.4)	0.8	0.8, 0.9
**Hospital admissions**									
Inpatient admissions^f^	288 (59.3)	2.0	1.8, 2.1	442 (90.9)	1.7	1.7, 1.8	361 (74.3)	3.4	3.2, 3.6
Admission (duration = 0 days)^g^	186 (38.3)	1.2	1.1, 1.3	379 (78.0)	1.2	1.1, 1.2	241 (49.6)	2.3	2.2, 2.5
Admission (duration ≥1 day)	213 (43.8)	0.7	0.6, 0.8	365 (75.1)	0.5	0.5, 0.5	267 (54.9)	1.1	1.0, 1.2
Readmissions within 30 days	68 (14.0)	0.2	0.2, 0.2	101 (20.8)	0.1	0.1, 0.1	50 (10.3)	0.2	0.1, 0.2
CC admissions^h^	18 (3.7)	0.0	0.0, 0.1	55 (11.3)	0.0	0.0, 0.0	28 (5.8)	0.1	0.1, 0.1

A&E, accident & emergency; CC, critical care; CI, confidence interval; EGPA, eosinophilic granulomatosis with polyangiitis; GP, general practitioner; HCRU, healthcare resource utilisation; HDU, high-dependency unit; ICU, intensive care unit; ID, index date; IM, intramuscular; IV, intravenous; PY, person-years.

a GP consultations included all consultations at a general practice, including those with a GP and those with a nurse.

b Drug codes for prescription cost analysis are presented in Supplemental Table 5. Biologics prescribed for the treatment of EGPA (mepolizumab, omalizumab, benralizumab, dupilumab, and rituximab) were not included, as these are predominantly prescribed and administered in the hospital setting and so are not well captured in the Clinical Practice Research Datalink Aurum database, which captures primary care data.

c Systemic glucocorticoids prescribed in primary care for the treatment of EGPA (oral prednisone, oral prednisolone, oral methylprednisolone, and IV/IM methylprednisolone).

d Immunosuppressants prescribed in primary care for the treatment of EGPA (azathioprine, cyclophosphamide, mycophenolate mofetil, and methotrexate).

e Only attended outpatient appointments were included.

f For patients with a diagnosis of EGPA recorded in Hospital Episodes Statistics, the admission pertaining to the diagnosis was allocated to the 0–12 month follow-up period.

g Admission with an admission date and a discharge date on the same day.

h CC included both ICU and HDU admissions

Among patients with inpatient hospitalisations lasting ≥1 day, the median length of stay was 5.0 (Q1–Q3: 2.0–11.0) days; for those with critical care admissions, the median stay was 2.0 (Q1–Q3: 1.0–7.0) days. Interestingly, although rates of inpatient admissions, admissions with discharge dates on the same day (duration = 0 days), and critical care admissions were similar in the 12 months prior to the ID and in the total post-ID period, they initially rose during the 12 months post-ID. In addition, outpatient visit rates were higher in the 12 months post-ID than in the total post-ID period.

**Fig. 1:**
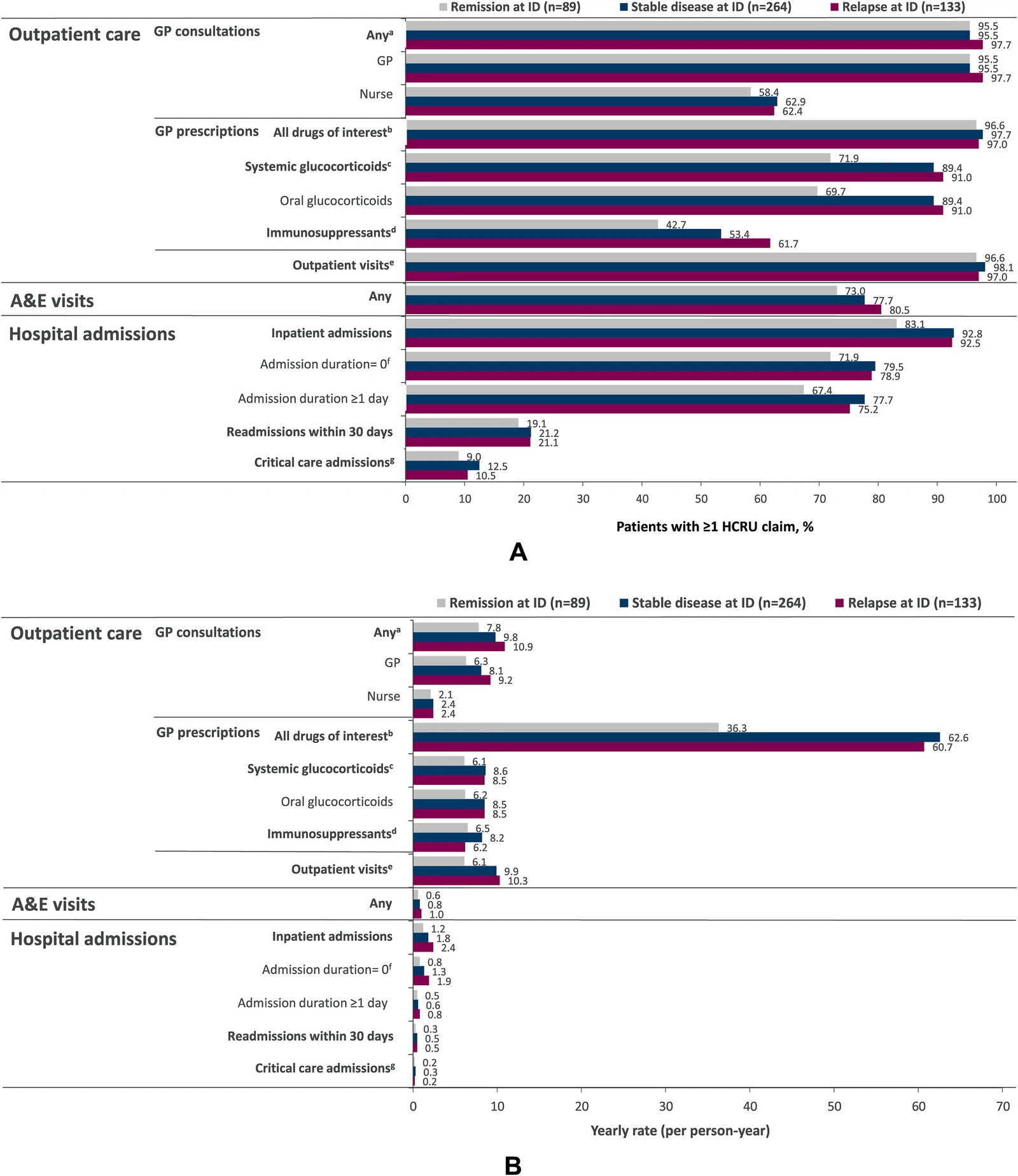
All-cause HCRU post-ID in the incident EGPA cohort by health state at ID: (A) percentage of patients with ≥1 HCRU claim, and (B) yearly rate (per person-year). ^a^GP consultations included all consultations at a general practice, including those with a GP and those with a nurse. ^b^Drug codes for prescription cost analysis are presented in Supplemental Table 5. ^c^Prescriptions for systemic glucocorticoids commonly used for the treatment of EGPA (oral prednisone, oral prednisolone, oral methylprednisolone, and intravenous/intramuscular methylprednisolone). ^d^Prescriptions for immunosuppressants commonly used in the treatment of EGPA (azathioprine, cyclophosphamide, mycophenolate mofetil, and methotrexate). ^e^Only attended outpatient appointments were included. ^f^Admission with an admission date and a discharge date on the same day. ^g^ICU and HDU admissions. EGPA, eosinophilic granulomatosis with polyangiitis; GP, general practitioner; HCRU, healthcare resource utilisation; HDU, high-dependency unit; ICU, intensive care unit; ID, index date

The all-cause HCRU post-ID in the incident EGPA cohort by health state at ID is summarised in Fig. 1. Patients with EGPA in remission exhibited the lowest annualised HCRU rates and the smallest proportions of A&E visits, prescriptions for systemic glucocorticoids and immunosuppressants, inpatient admissions, and inpatient admissions lasting either 0 or ≥1 day compared with those with stable disease or relapse (Fig. 1), while usage in other categories showed little to no difference across groups.

Patients with an FFS of 0 generally exhibited lower HCRU than those with an FFS ≥1 (Table 3).

**Table 3:** All-cause HCRU (total post-ID period) in the EGPA cohort by FFS 1996.

**Source of HCRU**	FFS 1996 = 0			FFS 1996 ≥ 1		
	**Patients with resource usage, n (%)**	**Rate per PY (all patients)**	**95% CI**	**Patients with resource usage, n (%)**	**Rate per PY (all patients)**	**95% CI**
**Number of patients at start of period**	**371**			**115**		
Outpatient care						
GP consultations^a^	362 (97.6)	9.2	9.1, 9.3	105 (91.3)	11.6	11.3, 11.9
GP	362 (97.6)	7.7	7.6, 7.8	105 (91.3)	9.4	9.1, 9.6
Nurse	238 (64.2)	1.5	1.4, 1.5	63 (54.8)	2.2	2.1, 2.4
GP prescriptions (all drugs of interest^b^)	363 (97.8)	53.3	53.0, 53.6	110 (95.7)	73.1	72.4, 73.9
Systemic glucocorticoids^c^	326 (87.9)	7.3	7.2, 7.4	95 (82.6)	8.6	8.3, 8.8
Immunosuppressants^d^	198 (53.4)	4.0	3.9, 4.0	63 (54.8)	5.5	5.3, 5.7
Outpatient visits^e^	364 (98.1)	9.0	8.8, 9.1	110 (95.7)	10.5	10.2, 10.8
A&E visits	290 (78.2)	0.6	0.6, 0.7	87 (75.7)	0.9	0.8, 0.9
Hospital admissions						
Inpatient admissions	332 (89.5)	1.5	1.4, 1.5	110 (95.7)	2.7	2.5, 2.8
Admission (duration = 0 days)^f^	291 (78.4)	1.0	0.9, 1.0	88 (76.5)	2.1	1.9, 2.2
Admission (duration ≥1 day)	277 (74.7)	0.5	0.5, 0.5	88 (76.5)	0.6	0.5, 0.7
Readmissions within 30 days	75 (20.2)	0.1	0.1, 0.1	26 (22.6)	0.1	0.1, 0.1
CC admissions^g^	40 (10.8)	0.0	0.0, 0.0	15 (13.0)	0.0	0.0, 0.1

A&E, accident & emergency; CC, critical care; CI, confidence interval; EGPA, eosinophilic granulomatosis with polyangiitis; FFS, Five-Factor Score; GP, general practitioner; HCRU, healthcare resource utilisation; HDU, high-dependency unit; ICU, intensive care unit; ID, index date; PY, person-years.

a GP consultations included all consultations at a general practice, including those with a GP and those with a nurse.

b Drug codes for prescription cost analysis are presented in Supplemental Table 5. Biologics prescribed for the treatment of EGPA (mepolizumab, omalizumab, benralizumab, dupilumab, and rituximab) were not included, as these are predominantly prescribed and administered in the hospital setting and so are not well captured in the Clinical Practice Research Datalink Aurum database, which captures primary care data.

c Prescriptions for systemic glucocorticoids commonly used for the treatment of EGPA (oral prednisone, oral prednisolone, oral methylprednisolone, and intravenous/intramuscular methylprednisolone).

d Prescriptions for immunosuppressants commonly used in the treatment of EGPA (azathioprine, cyclophosphamide, mycophenolate mofetil, and methotrexate).

e Only attended outpatient appointments were included.

f Admission with an admission date and a discharge date on the same day.

g CC included both ICU and HDU admissions

Median and mean annualised total all-cause costs in the incident EGPA cohort during the entire post-ID period were £6219 (Q1–Q3: £3272–£12,153) and £14,261 (SD: £43,916), respectively. Hospital admissions represented the largest contributor to these costs (53.5%), with a median of £3328 (Q1–Q3: £1398–£6686) and a mean of £11,365 (SD: £45,095). Outpatient care also accounted for a significant portion of total expenditures (33.8%), with median and mean costs of £2100 (Q1–Q3: £1159–£3412) and £2544 (SD: £2032), respectively. Costs associated with GP prescriptions and A&E visits were minor components, with estimated median costs of £348 (Q1–Q3: £198–£620) and £163 (Q1–Q3: £86–£392, respectively, alongside mean costs of £484 (SD: £458) and £361 (SD: £836), respectively.

**Fig. 2:**
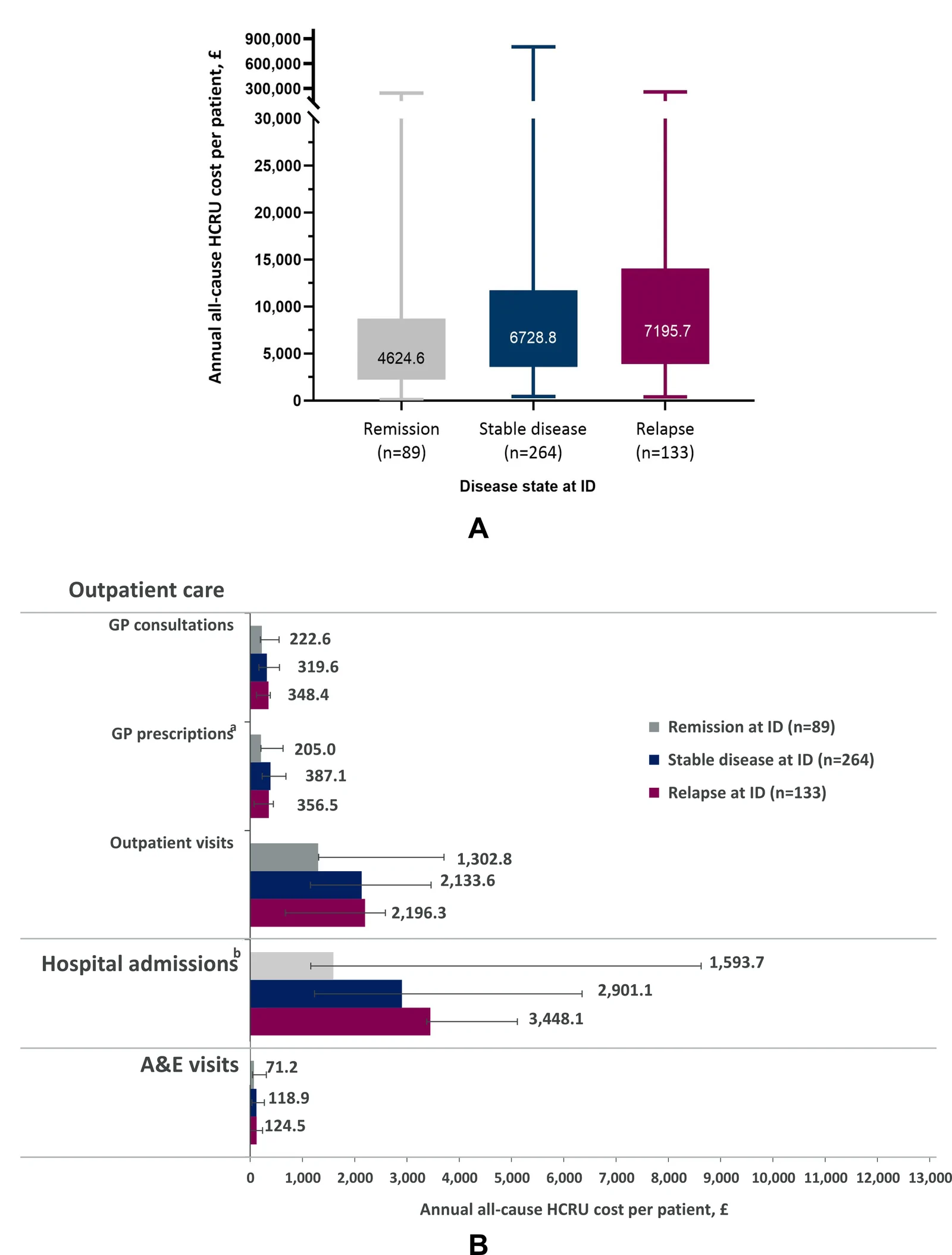
All-cause HCRU costs in the incident EGPA cohort by disease state at ID (median [Q1; Q3]) all-cause annualised costs per patient): (A) total and (B) by individual HCRU categories. ^a^Drug codes for prescription cost analysis are presented in Supplemental Table 5. ^b^Including ICU and HDU admissions. A&E, accident & emergency; EGPA, eosinophilic granulomatosis with polyangiitis; GP, general practitioner; HCRU, healthcare resource utilisation; HDU, high-dependency unit; ICU, intensive care unit; ID, index date

During follow-up, median all-cause annualised total costs per patient were 46% higher in patients with stable disease and 56% higher in those with relapse compared with patients in remission at ID (Fig. 2A). The greatest differences were seen for costs related to hospital admissions (£3448 for patients in relapse, £2901 for patients with stable disease, and £1594 for patients in remission; Fig. 2B).

### Comparison of HCRU in EGPA versus matched general population and SUA cohorts

**Fig. 3:**
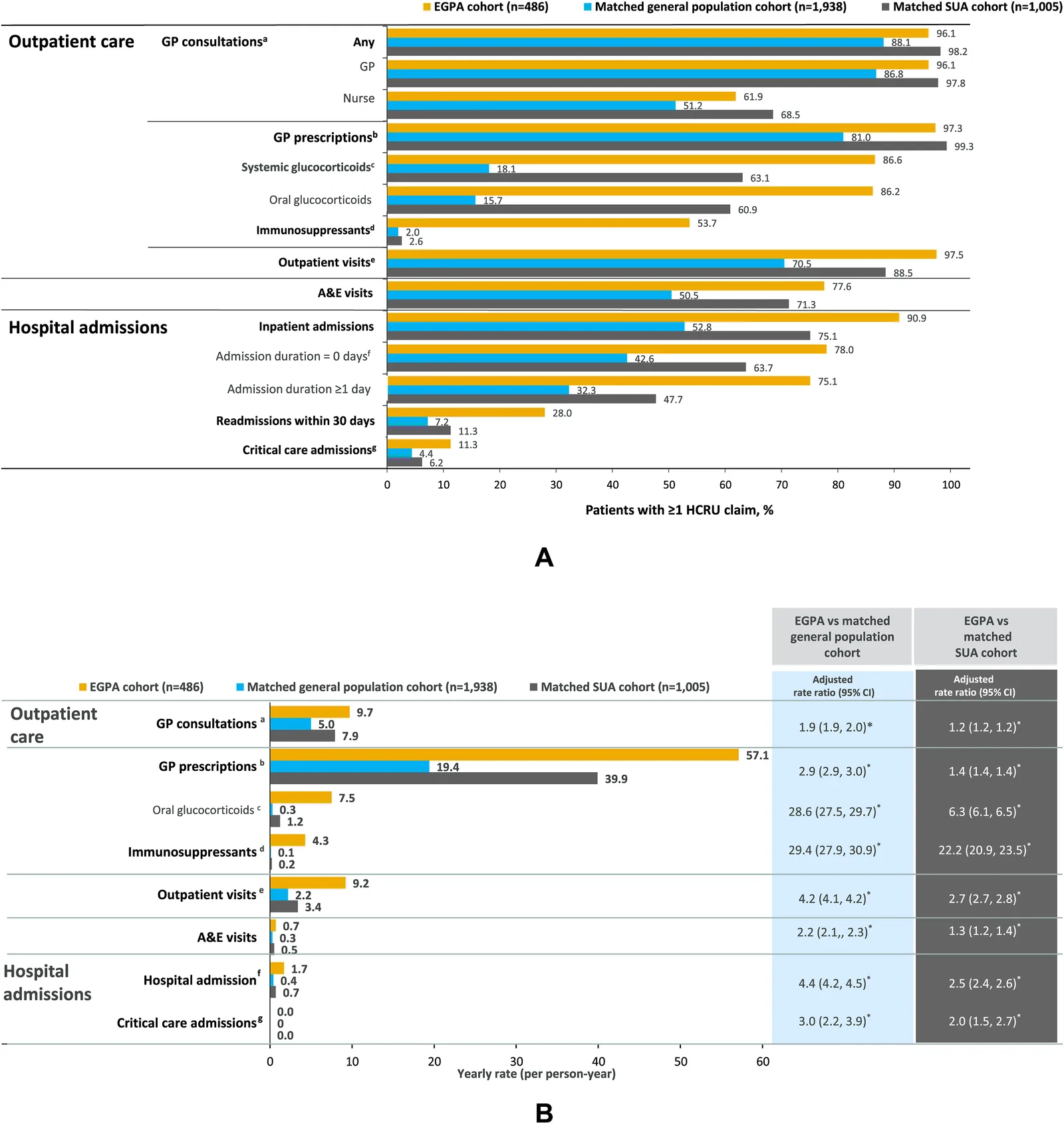
All-cause HCRU post-ID in the incident EGPA cohort compared with the matched general population and SUA cohorts: (A) percentage of patients with ≥1 HCRU claim, and (B) yearly rate (per person-year). ∗p < 0.001. ^a^GP consultations included all consultations at a general practice, including those with a GP and those with a nurse. ^b^Prescriptions for all drugs of interest; drug codes for prescription cost analysis are presented in Supplemental Table 5. ^c^Prescriptions for systemic glucocorticoids commonly used for the treatment of EGPA (oral prednisone, oral prednisolone, oral methylprednisolone, and intravenous/intramuscular methylprednisolone). ^d^Prescriptions for immunosuppressants commonly used in the treatment of EGPA (azathioprine, cyclophosphamide, mycophenolate mofetil, and methotrexate). ^e^Only attended outpatient appointments were included; ^f^Admission with an admission date and a discharge date on the same day. ^g^ICU and HDU admissions. A&E, accident and emergency; CI, confidence interval; EGPA, eosinophilic granulomatosis with polyangiitis; GP, general practitioner; HCRU, healthcare resource utilisation; HDU, high-dependency unit; ICU, intensive care unit; ID, index date; SUA, severe uncontrolled asthma

Throughout the total post-ID period, both the proportion of patients with HCRU and the annualised HCRU rates were substantially higher across all resource categories in the incident EGPA cohort compared with the matched general population cohort (Fig. 3 and Supplemental Table 2). Similarly, annualised HCRU rates were substantially higher across all resource categories in the incident EGPA cohort compared with the matched SUA cohort, except for intravenous (IV) or intramuscular (IM) glucocorticoids and critical care admissions, where low recorded usage precluded meaningful comparisons (Fig. 3 and Supplemental Table 3). The proportion of patients with general practice consultations overall (with a GP or nurse), GP prescriptions (for all drugs of interest), and A&E visits were similar between the incident EGPA and SUA cohorts, whereas the incident EGPA cohort had higher proportions of patients across all other HCRU categories.

**Fig. 4:**
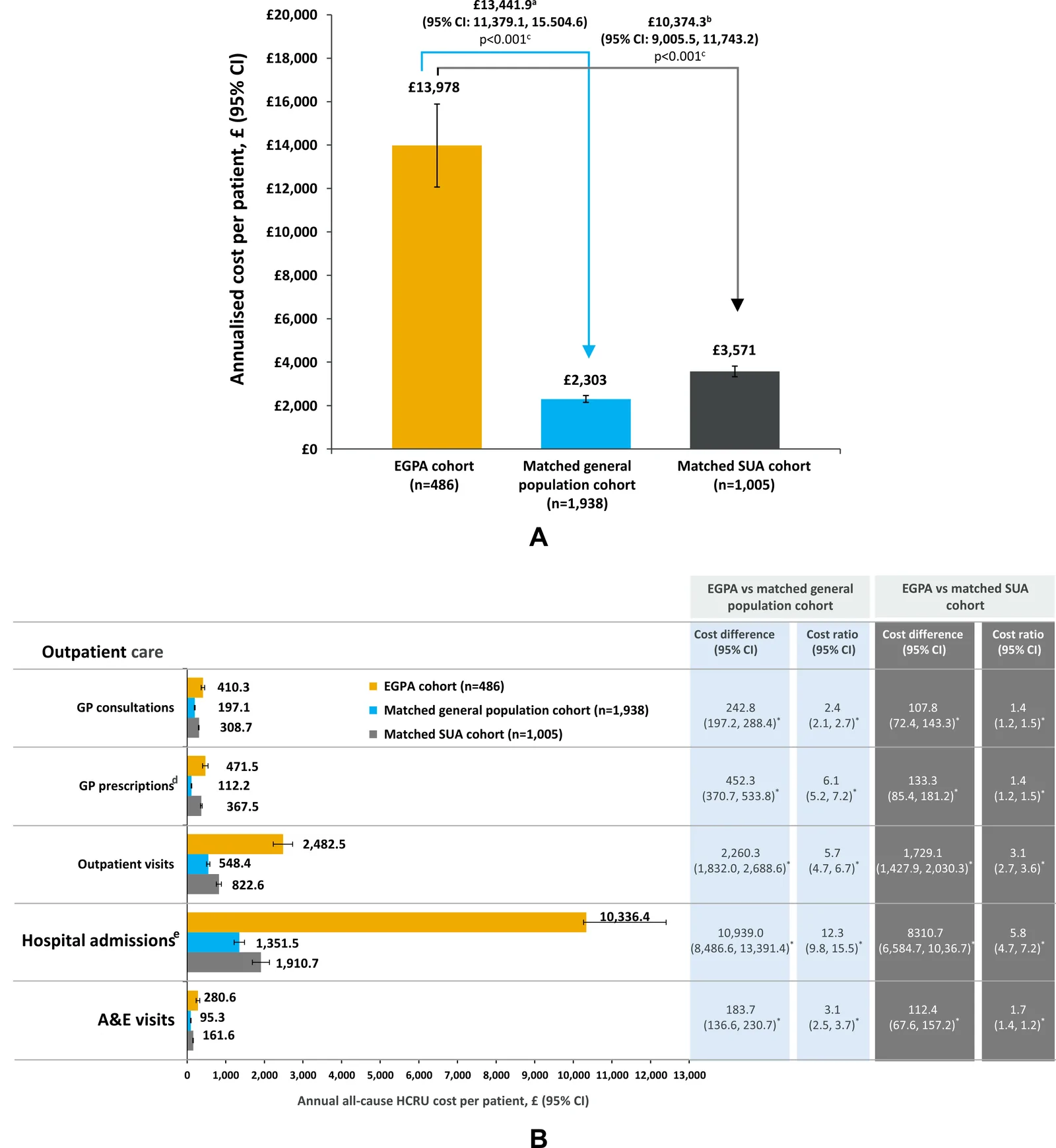
Multivariable analysis comparing all-cause mean annual costs in the EGPA cohort compared with the matched general population and SUA cohorts: (A) total and (B) by individual HCRU categories. ∗p < 0.001. ^a^Cost difference for the EGPA cohort versus the matched general population cohort. ^b^Cost difference for the EGPA cohort versus the matched SUA cohort. ^c^p-values from multivariable generalised linear regression model. ^d^All drugs of interest; drug codes for prescription cost analysis are presented in Supplemental Table 5. ^e^Including ICU and HDU admissions. A&E, accident & emergency; CI, confidence interval; EGPA, eosinophilic granulomatosis with polyangiitis; GP, general practitioner; HCRU, healthcare resource utilisation; HDU, high-dependency unit; ICU, intensive care unit; SUA, severe uncontrolled asthma

Across all HCRU categories, the incident EGPA cohort incurred higher annualised costs than the matched general population and SUA cohorts (Supplemental Table 4). Multivariable analyses demonstrated significantly higher total annualised mean all-cause costs across all HCRU categories in the EGPA cohort. Overall, costs were more than 8-fold higher than in the general population and over 4-fold higher in patients with SUA (Fig. 4A), with the greatest increase reported for costs related to hospital admissions (£10,336 in the EGPA cohort vs £1911 in the matched SUA cohort (cost ratio [95% CI]: 5.8 [4.7, 7.2]) vs £1351 in the matched general population cohort (cost ratio [95% CI]: 12.3 [9.8, 15.5]; Fig. 4B). Following hospital admissions, the next largest cost drivers were GP prescriptions (cost ratio [95% CI]: 6.1 [5.2, 7.2] and outpatient visits (cost ratio [95% CI]: 5.7 [4.7, 6.7]) for patients with EGPA compared with the matched general population cohort, and outpatient visits (cost ratio [95% CI]: 3.1 [2.7, 3.6]) followed by GP prescriptions (cost ratio [95% CI]: 1.4 [1.2, 1.5]) for patients with EGPA compared with the matched SUA cohort (Fig. 4B).

## Discussion

Our study is one of the largest and most comprehensive real-world analyses to date to quantify the clinical and economic burden of EGPA.^4^^,^^9^^,^^10^^,^^21^, ^22^, ^23^ While a recent retrospective study using the CPRD Aurum database also assessed EGPA-related HCRU over a similar time period,^4^ our analysis goes further by providing direct comparisons with matched cohorts from the general population without EGPA and patients with SUA, as well as by EGPA disease state. Patients with EGPA exhibited substantially higher HCRU and associated costs compared with the general population cohort, particularly during the first year following diagnosis. While the differences between the incident EGPA and SUA cohorts were less pronounced than those observed between the incident EGPA and general population cohorts, patients with EGPA still incurred consistently higher annualised HCRU rates and costs across all categories. In particular, annualised costs per-patient related to hospital admissions in the EGPA cohort were 12.3-fold higher than those in the general population cohort and 5.8-fold higher than those in the SUA cohort. Notably, patients with EGPA and an FFS of 0 generally demonstrated lower HCRU than those with an FFS ≥1.

Among patients with EGPA who utilised healthcare resources, median all-cause annualised costs were approximately 8-fold higher than those in the matched general population cohort, and approximately 4-fold higher than those in the SUA cohort. These findings are consistent with previous research. A recent US-based retrospective cohort study using a managed care database reported mean all-cause costs that were approximately twice as high in the EGPA cohort compared with a matched asthma cohort.^9^ Similarly, another US analysis of health insurance claims data found that all-cause healthcare costs were approximately 16 times higher in patients with EGPA than in those in a matched general insured population cohort, and approximately 5 times higher than those in a matched SUA cohort. In Europe, a study analysing claims data from the German AOK health insurance database reported that all-cause annualised HCRU costs were 7-fold higher in the EGPA cohort than those in the matched general insured patient cohort.^22^ Across all these studies ‒ including the current analysis ‒ inpatient hospitalisations consistently emerged as the primary driver of overall healthcare costs in patients with EGPA.^9^^,^^21^^,^^23^ These findings also underscore the clinical heterogeneity of EGPA and the potential value of further characterisation and stratification by concomitant asthma status in future research. Although asthma is a key clinical feature of EGPA,^2^^,^^5^ it varies in both severity and level of control;^24^^,^^25^ consequently, patients with asthma may exhibit distinct patterns of HCRU and associated costs compared with those without asthma. However, as asthma ascertainment in CPRD Aurum was based on recorded diagnostic codes, some degree of misclassification cannot be excluded, particularly given the well-recognised under- and over-diagnosis of asthma in routine clinical practice. Future studies using datasets with more comprehensive clinical characterisation may enable more robust stratified analyses by asthma status, helping to disentangle the relative contributions of vasculitic and respiratory disease components from HCRU, thereby generating more granular evidence to inform tailored management strategies.

Patients classified as being in remission at ID incurred lower HCRU and associated costs than those in stable disease or experiencing relapse. Median annualised total costs were approximately 1.5 times higher in patients with stable disease or relapse than in those in remission, with the most notable differences observed in hospital admissions. These data highlight the economic impact of achieving remission in EGPA, underscoring the importance of strategies that not only prevent relapse but enable patients to achieve a state of no disease activity and low doses of glucocorticoids, instead of aiming for mere disease stabilisation.

These findings also align with 2 of the previously mentioned US studies, which reported substantially elevated follow-up costs among patients with major active disease at baseline; in both, costs for those with major active disease approximately tripled over the follow-up period compared with those with remission and those with non-major active disease, for which costs approximately doubled during the respective follow-up periods.^21^^,^^23^ Similarly, in the third US-based analysis, mean all-cause costs were approximately 3.5-fold higher in EGPA patients with a relapse requiring hospitalisation than costs in those without evidence of relapse.^9^

Recently published guidelines from the British Society for Rheumatology recommend the use of anti-interleukin-5/R biologics for the induction of remission,^26^ in line with US guidelines and broadly aligned with European guidelines.^5^^,^^26^^,^^27^ Although in the United Kingdom, access to biologic therapies for EGPA patients has traditionally been restricted to those with concomitant severe asthma, this landscape is evolving. In light of positive results from randomised controlled trials for mepolizumab^28^ and benralizumab,^29^ the National Institute for Health and Care Excellence has recently issued a positive recommendation for benralizumab as an add-on treatment for adults with relapsing or refractory EGPA.^30^ Notably, the majority (79.8%) of patients in the incident EGPA cohort had comorbid asthma; however, biologic therapies were not included in this analysis, as they are predominantly prescribed and administered in specialist settings and are therefore not well captured in CPRD Aurum, which primarily captures primary care data. This limitation may have also impacted the overall calculations for outpatient visits. Consequently, biologic use was likely under-captured, precluding meaningful analyses of the impact of biologic therapies on hospitalisation costs within this study.

Another limitation of our study is the fact that the database does not capture information on the reasons for drug prescriptions, such as whether they were for EGPA or a comorbid condition; therefore, accurately determining the true extent of HCRU and the associated cost burden attributable specifically to EGPA is challenging in this analysis. In addition, the data for the FFS subgroup analysis were estimated using study-specific algorithms based on proxy codes for the FFS 1996 version components; however, these algorithms have not been previously validated. As such, it was not possible to determine whether patients were receiving chronic drug treatment as maintenance therapy or for the management of a relapse. Health states (remission, stable disease, and relapse) were categorised primarily based on EGPA manifestations, hospitalisations, and glucocorticoid prescriptions, rather than physician-assigned diagnoses; as such, these classifications may be subject to misclassification or inaccuracies. Nevertheless, the use of the CPRD Aurum database represents a key strength of the study due to its large size, broad coverage, and representativeness of the primary care population in England, along with its longitudinal design, standardised linkages to other healthcare datasets, and robust data quality assurance.^31^

In conclusion, our study highlights the substantial HCRU and cost burden associated with EGPA in England, underscoring a significant unmet need in clinical practice. Novel treatments that enable patients to achieve and sustain remission have the potential not only to directly improve patient outcomes but also to alleviate clinical and financial pressure on healthcare systems.

## Consent to publish

All authors reviewed and revised the manuscript critically and approved the final version for submission. All authors had access to the study data.

## Author contributions

SHS, PD, SYC, CE and JR contributed to the study conceptualisation and methodology. AL, BD, PS-S, CA, BP and AKV contributed to data curation, formal analysis, methodology and validation. All authors contributed to data interpretation, as well as writing (reviewing and editing).

## Ethics statement

The protocol (22_001934) was approved by an Independent Scientific Advisory Committee. The data, provided by patients and collected by the NHS as part of their care and support, was obtained from the Clinical Practice Research Datalink under licence from the UK Medicines and Healthcare products Regulatory Agency.

## Editorial policy confirmation and agreement

The research described in this article has not been published previously, it is not under consideration for publication elsewhere, and its publication is approved by all authors and tacitly or explicitly by the responsible authorities where the work was carried out. If accepted, this work will not be published elsewhere in the same form, in English or in any other language, including electronically without the written consent of the copyright holder.

## Data sharing statement

This study is based in part on data from the Clinical Practice Research Datalink obtained under licence from the UK Medicines and Healthcare products Regulatory Agency, and are therefore not publicly available. The data is provided by patients and collected by the NHS as part of their care and support. The interpretation and conclusions contained in this study are those of the author/s alone.

## Use of generative artificial intelligence (AI) and AI-assisted technologies

Nothing to disclose.

## Role of the funding source

The sponsor had a role in the study design; in the collection, analysis, and interpretation of data; in the writing of the report, and in the decision to submit the article for publication.

## Funding

This study was funded by AstraZeneca.

## Declaration of interest statement

SHS has received speaker fees from GSK, AstraZeneca, Boehringer Ingelheim, Chiesi&Medscape and Novartis; participates on advisory boards for AstraZeneca, Chiesi, GSK, Areteia Therapeutics, Kymera Therapeutics, and Lilly; has received grant funding from AstraZeneca and GSK.

BD, PD, CE, PJ, JR, LW and SYC are or were employees or contractors of AstraZeneca at the time of study conduct and may own stock/stock options.

AL, PS-S, CA, BP and AKV are employees of OXON Epidemiology, which received funding from AstraZeneca to conduct the study.
