# Supplementary material for: Healthcare resource utilisation and associated costs in patients with eosinophilic granulomatosis with polyangiitis in England: A retrospective observational cohort study
Source: World Allergy Organ J. 2026 Sep 2;19(9):101457. doi: 10.1016/j.waojou.2026.101457 (PMC13571310; doi:10.1016/j.waojou.2026.101457)
Supplement: Supplementary file 1 [file mmc1.docx]

# Supplemental Methods

## Supplemental Methods 1 Definition of matched general population and severe uncontrolled asthma (SUA) cohorts

**Matched general population cohort definition**

Patients were included in the matched general population cohort if they:

- Had no diagnosis record of eosinophilic granulomatosis with polyangiitis (EGPA) at any time.
- Were alive at the matched index date (ID) of the patient with EGPA, with at least 12 months look-back period before and inclusive of the ID.

A cohort of patients from the Clinical Practice Research Datalink (CPRD) Aurum database were selected by frequency matching on year of birth, sex, and general practitioner (GP) practice to patients in the incident EGPA cohort. Each patient with EGPA was matched with up to four persons from the general population cohort.

**Matched SUA cohort definition**

Patients were included in the matched SUA cohort if they had:

- At least 1 asthma diagnosis code in the CPRD database on or after 1 January 2006.
- At least 1 criterion from Part A, followed by at least 1 criterion from Part B, then at least 1 criterion from Part C, as identified through GP visits (CPRD Aurum).

**Part A (persistent):**

- At least 4 GP asthma diagnosis records plus at least 2 prescriptions for asthma control medications (inhaled corticosteroids [ICS] as monotherapy or in combination (ICS + long-acting β-agonist [LABA], ICS + LABA + long-acting muscarinic antagonist [LAMA]), leukotriene receptor antagonists (LTRAs) or theophylline in 1 year, OR
- At least 1 GP asthma diagnosis record, plus at least 4 prescriptions for any asthma control medication in 1 year.

**Part B (severe):**

- Met criteria of part A.
- Receipt of medium-to-high doses of ICS (monotherapy), ICS + LABA, or ICS + LABA + LAMA according to the Global Initiative for Asthma (GINA) guidelines,^11^ OR
- ≥180 cumulative day supply of oral glucocorticoids (OGCs; gaps present between prescription/repeats that total ≥180 days).

**Part C (uncontrolled):**

- Met criteria of part B.
- At least 2 GP visits for asthma followed by treatment with systemic glucocorticoids within a week (one dose of intravenous [IV] glucocorticoids or OGCs for at least three days), OR
- At least 4 prescriptions for a short-acting β-agonist (SABA) or SABA combinations within any 12-month period.

A cohort of patients with SUA were selected by frequency matching on year of birth, sex, and GP practice to patients in the incident EGPA cohort. Each patient with EGPA was matched with up to four persons from the SUA cohort.

## Supplemental Methods 2 Definitions of disease state

Patients were categorised into one of three mutually exclusive categories:

| **Disease state** | **Definition** |
| --- | --- |
| **Remission** | - No new EGPA manifestation, no OGC use or OGC use with a dose ≤4 mg/day, no initiation of an immunosuppressant or cyclophosphamide, and no EGPA-related hospitalisation. |
| **Relapse** | - New EGPA manifestation or EGPA-related hospitalisation, any systemic glucocorticoid initiation, re-initiation of >4mg/day or dose increase to >4mg/day, or initiation of an immunosuppressant or a cyclophosphamide prescription. - Type of relapses included asthma, nasal polyposis, vasculitis, and EGPA hospitalisation. |
| **Stable disease** | - Not in remission and not in relapse (e.g., all OGC doses above 4mg/day or only last dose of the time period below 4 mg/day). |

EGPA, eosinophilic granulomatosis with polyangiitis; OGC, oral glucocorticoid.

## Supplemental Methods 3 Calculation of Five Factor Score 1996

Five Factor Score (FFS) 1996 version^2^ scores of 0 or ≥ 1 were assigned using code proxies (Systematized Nomenclature of Medicine – Clinical Terms [SNOMED CT] or International Classification of Diseases, Tenth Revision, Clinical Modification [ICD-10-CM] codes) for each of the components listed below, with a lookback period of up to 5 years prior to index date (ID). An FFS score of 0 was assigned if no factors were present, a score of 1 if one factor was present, and a score of 2 if ≥2 factors were present.

- Proteinuria
- Creatininemia
- Severe gastrointestinal (GI) involvement
  - Acute pancreatitis
  - Chronic pancreatitis
  - GI bleed or perforation
  - GI ischaemia or infarction
- Cardiomyopathy
- Central nervous system (CNS) involvement
  - Cerebrovascular accident
  - Meningitis
  - Organic confusion
  - Seizure

As only 36% of the incident eosinophilic granulomatosis with polyangiitis (EGPA) cohort were over 65 years of age at ID, the 1996 version of the FSS was used instead of the 2009 version. The latter includes a component of age >65 years in its criteria, which would have limited its variability and utility in our cohort.

**ICD-10-CM and SNOMED CT codes for the components of the FFS 1996 version:**

| **Characteristic** | **ICD-10-CM** | **SNOMED CT** |
| --- | --- | --- |
| **Proteinuria** |  |  |
| Isolated proteinuria | R80 | 12491000132101 |
| Persistent proteinuria, unspecified | N39.1 | 12511000132108 |
| **Creatininemia^a^** |  |  |
| Stage IIIb–V chronic kidney disease, end stage renal disease, or dialysis | I12.0, I13.11, I13.2, N18.32, N18.4, N18.5, N18.6, Z49, Z99.2 | 236436003, 236435004, 46177005, 302497006, 324371000000106, 324411000000105, 700379002, 433146000, 265764009, 431857002, 129151000119102, 721000119107, 324441000000106, 324471000000100, 90761000119106, 711000119100, 714152005, 324501000000107, 714153000, 324541000000105, 714749008, 233581009, 71192002, 42399005, 90688005, 950061000000103, 950081000000107, 950231000000104, 950101000000101, 950181000000106, 950311000000102, 950251000000106, 950291000000103, 950211000000107 |
| **Severe GI involvement:**  Bowel perforation or bleeding (not due to appendicitis or cholecystitis) | K20.81, K20.91, K21.01, K22.11, K22.3, K25.0, K25.1, K25.2, K25.4, K25.5, K25.6, K26.0, K26.1, K26.2, K26.4, K26.5, K26.6, K27.0, K27.1, K27.2, K27.4, K27.5, K27.6, K28.0, K28.1, K28.2, K28.4, K28.5, K28.6, K29.01, K29.21, K29.31, K29.41, K29.51, K29.61, K29.71, K29.81, K29.91, K31.811, K35.2, K35.32, K35.33, K50.011, K50.111, K50.811, K50.911, K51.011, K51.211, K51.311, K51.411, K51.511, K51.811, K51.911, K55.21, K57.0, K57.11, K57.13, K57.2, K57.31, K57.33, K57.4, K57.51, K57.53, K57.8, K57.91, K57.93, K62.5, K63.1, K92.0, K92.1, K92.2 | 786878009, 6072007, 786878009, 38851006, 308904008, 235671002, 74474003, 37831005, 51875005, 38851006, 56905009, 87763006, 307297004, 722886001, 30873000, 23387001, 48729005, 235674005, 307296008, 37372002, 8765009, 40835002, 267051003, 2901004, 414663001, 15902003, 9829001, 39204006, 13200003, 51868009, 16121001, 13200003, 27281001, 88968005, 64121000, 88169003 |
| GI infarction or ischaemia | K55.0, K55.1, K55.8, K55.9 | 111354009, 22323009, 235842000, 27241008, 30588004, 3558002, 46882000, 75700000, 82196007, 906191000000102, 91489000, 312607004, 241154007, 734041006 |
| Pancreatitis (acute or chronic) | K85.0, K85.1, K85.9, K86.1 | 197456007, 4399003, 7881005, 197458008, 811431000000107, 235943006, 235494005, 235953007, 235954001, 301009006, |
| **Cardiomyopathy** | I25.5, I42.0, I42.1, I42.2, I42.3, I42.4, I42.5, I42.7, I42.8, I42.9, I43, O90.3 | 195029002, 233873004, 399020009, 415295002, 426300009, 441541008, 471870000, 62377009, 64077000, 85898001, 195021004, 45227007, 194849004, 195020003, |
| **CNS involvement** |  |  |
| Cerebrovascular accident | I60, I60.0, I60.1, I60.2, I60.3, I60.4, I60.5, I60.6, I60.7, I60.8, I60.9, I61, I61.0, I61.1, I61.2, I61.3, I61.4, I61.5, I61.6, I61.8, I61.9, I62, I62.0, I62.1, I62.9, I63, I63.0, I63.1, I63.2, I63.3, I63.4, I63.5, I63.6, I63.8, I63.9, I64, I67.6, I69.0, I69.1, I69.2, I69.3, I69.4, I69.8 | 230690007, 195212005, 16371781000119100, 788880006, 788882003, 788883008, 788884002, 230691006, 16023991000119104, 16000431000119109, 16000391000119104, 16000511000119103, 16000351000119109, 329361000119107, 16644681000119102  63230211000119103, 137592291000119101, 272202251000119109, 407573811000119100  502836591000119106, 738478141000119101, 788310011000119109, 16661971000119104  46583221000119102, 693158221000119105  595899961000119100, 652287331000119104, 25133001, 371041009, 281240008,  1078001000000105, 422504002, 39925003  195216008, 111297002, 195217004, 275434003  230713003, 371040005 |
| Meningitis | G00, G00.0, G00.1, G00.2, G00.3, G00.8, G00.9, G01  G02, G02.0, G02.1, G02.8, G03, G03.0, G03.1, G03.2, G03.8, G03.9 | 7180009, 822031000000101, 301770000, 21664006, 312216007, 192679007  230154004, 81981006 |
| Organic confusion | F05, F05.0, F05.1, F05.8, F05.9 | 2776000, 191505005, 191504009, 280994000  725898002, 231440006, 10349009, 191507002 |
| Seizure | R56.8, G40, G40.0, G40.1, G40.2, G40.3, G40.4, G40.5, G40.6, G40.7, G40.8, G40.9, G41, G41.0, G41.1, G41.2, G41.8, G41.9 | 443410001, 426634003, 53482009, 290871000119101, 241006, 83752007, 41510006, 58895005, 444229001  277130004, 13973009, 230456007  66264000, 91175000, 128613002, 313307000  703150000, 770758009, 84757009 |

^a^For creatininemia other codes were included: HCPCS (G0049, G0052, G0257, G2170, G2171) and CPT (36818, 16819, 36820, 36825, 35830, 90935, 90937, 90945, 90947, 90960, 90961, 90962, 90963, 90966, 90970).
CNS, central nervous system; CPT, current procedural terminology; GI, gastrointestinal; HCPCS, healthcare common procedure coding system; ICD-10-CM, International Classification of Diseases, Tenth Revision, Clinical Modification.

## Supplemental Methods 4 Healthcare resource reference units

| Resource | **Unit** | **Reference** | **Cost – key variable** |
| --- | --- | --- | --- |
| GP consultations (including those with GPs and nurses) | GP with qualification costs: Per surgery consultation lasting 9.22 minutes, including direct care staff costs | PSSRU 2022 - Table 9.4.2: Unit costs for a GP | £41.00 |
|  | Nurse with qualification costs: Per surgery consultation lasting 15.5 minutes, including direct care staff costs | PSSRU 2022 - Table 9.3.1: Costs and unit estimations for nurses working in a GP practice nurse (Band 5)  PSSRU 2015 - Table 10.6: Duration of contact | £13.43 |
| GP prescriptions (all drugs of interest) | As per lookup table ‘drugscost’ | Prescription cost analysis calendar year 2022; Prescription cost analysis Financial year 2022–2023 > National tariff VIIIA December 2022 > Manual imputation (dmd Browser or OpenPrescribing) > estnhscost | Item cost |
| Outpatient visits | Unit cost of outpatient procedures | National cost collection: National schedule of NHS costs - Year 2021/22 - NHS trusts and NHS foundation trusts - Outpatient procedures | £229.00 |
| A&E visits | Unit cost of emergency care | National cost collection: National schedule of NHS costs - Year 2021/22 - NHS trusts and NHS foundation trusts - Emergency care | £242.03 |
| Hospital admissions | Unit cost of elective inpatient stays | National cost collection: National schedule of NHS costs - Year 2021/22 - NHS trusts and NHS foundation trusts - Elective inpatients | £5845.22 |
|  | Unit cost of non-elective inpatient stays (long stays) | National cost collection: National schedule of NHS costs - Year 2021/22 - NHS trusts and NHS foundation trusts - Non-elective inpatient - Long stay | £4408.91 |
|  | Unit cost of non-elective inpatient stays (short stays) | National cost collection: National schedule of NHS costs - Year 2021/22 - NHS trusts and NHS foundation trusts - Non-elective inpatient - Short stay | £801.11 |
|  | Unit cost of day cases | National cost collection: National schedule of NHS costs - Year 2021/22 - NHS trusts and NHS foundation trusts - Day case | £1038.36 |
|  | Unit cost of regular day or night admission | National cost collection: National schedule of NHS costs - Year 2021/22 - NHS trusts and NHS foundation trusts - Regular day or night admissions | £405.78 |
| Critical care admissions | Unit cost of critical care | National cost collection: National schedule of NHS costs - Year 2021/22 - NHS trusts and NHS foundation trusts - Critical care | £1752.87 |

A&E, accident and emergency; GP, general practitioner; NHS, National Health Service; PSSRU, Personal Social Services Research Unit.

# Supplemental tables

## Supplemental Table 1 Sociodemographic characteristics at ID in the matched general population and SUA cohorts

|  | Matched general population cohort (n=1,938) | Matched SUA cohort (n=1,005) |  |
| --- | --- | --- | --- |
| **Age** |  |  |  |
| Mean (SD) | 57.8 (15.1) | 58.5 (13.5) |  |
| Median (Q1–Q3) | 60.0 (48.0–69.0) | 60.0 (49.0–68.0) |  |
| Min; Max | 12.0; 91.0 | 12.0; 86.0 |  |
| Categorical, n (%) |  |  |  |
| <35 years^a^ | 144 (7.4) | 40 (4.0) |  |
| 35-49 years | 404 (20.8) | 230 (22.9) |  |
| 50-64 years | 692 (35.7) | 366 (36.4) |  |
| 65-79 years | 575 (29.7) | 317 (31.5) |  |
| 80+ years | 123 (6.3) | 52 (5.2) |  |
| **Sex, n (%)** |  |  |  |
| Male  Female  **Region, n (%)** | 965 (49.8)  973 (50.2) | 443 (44.1)  562 (55.9) |  |
| Northeast | 52 (2.7) | 26 (2.6) |  |
| Northwest | 399 (21.1) | 211 (21.0) |  |
| Yorkshire & the Humber | 56 (3.0) | 24 (2.4) |  |
| East Midlands | 16 (0.8) | 18 (1.8) |  |
| West Midlands | 312 (16.5) | 185 (18.4) |  |
| East of England | 128 (6.8) | 84 (8.4) |  |
| London | 327 (17.3) | 114 (11.3) |  |
| Southeast | 420 (22.2) | 222 (22.1) |  |
| Southwest | 184 (9.7) | 121 (12.0) |  |
| **Ethnicity, n (%)** |  |  |  |
| White | 1,348 (89.6) | 887 (91.1) |  |
| Asian | 70 (4.7) | 44 (4.5) |  |
| Black | 51 (3.4) | 27 (2.8) |  |
| Mixed/Other^b^ | 35 (2.3) | 16 (1.6) |  |
| Missing | 434 | 31 |  |
| **Index of Multiple Deprivation score, n (%)** |  |  |  |
| Quintile 1 (least deprived) | 440 (23.5) | 198 (19.7) |  |
| Quintile 2 | 406 (21.7) | 233 (23.2) |  |
| Quintile 3 | 363 (19.4) | 199 (19.8) |  |
| Quintile 4 | 357 (19.1) | 210 (20.9) |  |
| Quintile 5 (most deprived) | 304 (16.3) | 164 (16.3) |  |

^a^The <18 years (which had <5 patients) and 18–34 years age groups were combined due to low patient numbers. ^b^The Mixed ethnicity group was combined with the Other ethnicity group due to low patient numbers. ^c^Study recruitment concluded on 28 February 2019, and follow-up continued until 28 February 2020.

ID, index date; SD, standard deviation; SUA, severe uncontrolled asthma.

## Supplemental Table 2 All-cause HCRU usage over time in the matched general population cohort compared with the incident EGPA cohort

|  | Matched general population cohort | | | | | Incident EGPA cohort | | |
| --- | --- | --- | --- | --- | --- | --- | --- | --- |
| Source of HCRU | Patients with  resource usage, n (%) | Rate per PY  (all patients) | 95% CI | | Patients with  resource usage, n (%) | | Rate per PY  (all patients) | 95% CI |
| **Outpatient care** |  |  |  | |  | |  |  |
| GP consultations^a^ | 1,707 (88.1) | 5.0 | 4.9, 5.0 | | 467 (96.1) | | 9.7 | 9.5, 9.8 |
| GP | 1,683 (86.8) | 4.3 | 4.2, 4.3 | | 467 (96.1) | | 8.0 | 7.9, 8.1 |
| Nurse | 992 (51.2) | 0.7 | 0.7, 0.7 | | 301 (61.9) | | 1.6 | 1.6, 1.7 |
| GP prescriptions (All drugs of interest^b^) | 1,569 (81.0) | 19.4 | 19.3, 19.5 | | 473 (97.3) | | 57.1 | 56.8, 57.3 |
| Systemic glucocorticoids^c^ | 350 (18.1) | 0.3 | 0.3, 0.3 | | 421 (86.6) | | 7.5 | 7.4, 7.7 |
| Oral glucocorticoids | 304 (15.7) | 0.3 | 0.3, 0.3 | | 419 (86.2) | | 7.5 | 7.4, 7.6 |
| IV/IM glucocorticoids | 74 (3.8) | 0.0 | 0.0, 0.0 | | 15 (3.1) | | 0.0 | 0.0, 0.0 |
| Immunosuppressants^d^ | 38 (2.0) | 0.1 | 0.1, 0.2 | | 261 (53.7) | | 4.3 | 4.2, 4.3 |
| Outpatient visits^e^ | 1,366 (70.5) | 2.2 | 2.2, 2.2 | | 474 (97.5) | | 9.2 | 9.1, 9.4 |
| **A&E visits** | 979 (50.5) | 0.3 | 0.3, 0.3 | | 377 (77.6) | | 0.7 | 0.7, 0.7 |
| **Hospital admissions** |  |  |  |  |  | |  |  |
| Inpatient admissions | 1,024 (52.8) | 0.4 | 0.4, 0.4 | | 442 (90.9) | | 1.7 | 1.7, 1.8 |
| Admission (duration = 0 days)^f^ | 826 (42.6) | 0.2 | 0.2, 0.3 | | 379 (78.0) | | 1.2 | 1.1, 1.2 |
| Admission (duration ≥ 1 day) | 626 (32.3) | 0.1 | 0.1, 0.2 | | 365 (75.1) | | 0.5 | 0.5, 0.5 |
| Readmissions within 30 days | 139 (7.2) | 0.0 | 0.0, 0.0 | | 101 (20.8) | | 0.1 | 0.1, 0.1 |
| CC admissions^g^ | 86 (4.4) | 0.0 | 0.0, 0.0 | | 55 (11.3) | | 0.0 | 0.0, 0.0 |

^a^GP consultations included all consultations at a general practice, including those with a GP and those with a nurse. ^b^Drug codes for prescription cost analysis are presented in **Supplemental Table 5**. Biologics prescribed for the treatment of EGPA (mepolizumab, omalizumab, benralizumab, dupilumab, and rituximab) were not included, as these are predominantly prescribed and administered in the hospital setting and so are not well captured in the Clinical Practice Research Datalink Aurum database, which captures primary care data. ^c^Prescriptions for systemic corticosteroids commonly used in the treatment of EGPA (oral prednisone, oral prednisolone, oral methylprednisolone, and IV/IM methylprednisolone). ^d^Prescriptions for immunosuppressants commonly used in the treatment of EGPA (azathioprine, cyclophosphamide, mycophenolate mofetil, and methotrexate. ^e^Only attended outpatient appointments were included. ^f^Admission with an admission date and a discharge date on the same day. ^g^CC included both ICU and HDU admissions.

A&E, accident & emergency; CC, critical care; CI, confidence interval; EGPA, eosinophilic granulomatosis with polyangiitis; GP, general practitioner; HCRU, healthcare resource utilisation; HDU, high-dependency unit; ICU, intensive care unit; IM, intramuscular; IV, intravenous; PY, person-year.

## Supplemental Table 3 All-cause HCRU usage over time in the matched SUA cohort compared with the incident EGPA cohort

|  | | Matched SUA cohort | | | | | | Incident EGPA cohort | | | | |
| --- | --- | --- | --- | --- | --- | --- | --- | --- | --- | --- | --- | --- |
| Source of HCRU | | Patients with  resource usage, n (%) | | Rate per PY  (all patients) | | 95% CI | | Patients with  resource usage,  n (%) | Rate per PY  (all patients) | | 95% CI | |
| **Outpatient care** | |  | |  | |  | |  |  | |  | |
| GP consultations^a^ | | 987 (98.2) | | 7.9 | | 7.9, 8.0 | | 467 (96.1) | 9.7 | | 9.5, 9.8 | |
| GP | | 983 (97.8) | | 6.5 | | 6.4, 6.6 | | 467 (96.1) | 8.0 | | 7.9, 8.1 | |
| Nurse | | 688 (68.5) | | 1.4 | | 1.4, 1.5 | | 301 (61.9) | 1.6 | | 1.6, 1.7 | |
| GP prescriptions (All drugs of interest^b^) | | 998 (99.3) | | 39.9 | | 39.8, 40.1 | | 473 (97.3) | 57.1 | | 56.8, 57.3 | |
| Systemic glucocorticoids^c^ | | 634 (63.1) | | 1.2 | | 1.2, 1.2 | | 421 (86.6) | 7.5 | | 7.4, 7.7 | |
| Oral glucocorticoids | | 612 (60.9) | | 1.2 | | 1.2, 1.2 | | 419 (86.2) | 7.5 | | 7.4, 7.6 | |
| IV/IM glucocorticoids | | 74 (7.4) | | 0.0 | | 0.0, 0.0 | | 15 (3.1) | 0.0 | | 0.0, 0.0 | |
| Immunosuppressants^d^ | | 26 (2.6) | | 0.2 | | 0.2, 0.2 | | 261 (53.7) | 4.3 | | 4.2, 4.3 | |
| Outpatient visits^e^ | | 889 (88.5) | | 3.4 | | 3.4, 3.5 | | 474 (97.5) | 9.2 | | 9.1, 9.4 | |
| **A&E visits** | | 717 (71.3) | | 0.5 | | 0.5, 0.5 | | 377 (77.6) | 0.7 | | 0.7, 0.7 | |
| **Hospital admissions** |  |  |  | |  | |  | | |  | |  |
| Inpatient admissions | | 755 (75.1) | | 0.7 | | 0.7, 0.7 | | 442 (90.9) | 1.7 | | 1.7, 1.8 | |
| Admission (duration = 0 days)^f^ | | 640 (63.7) | | 0.4 | | 0.4, 0.5 | | 379 (78.0) | 1.2 | | 1.1, 1.2 | |
| Admission (duration ≥ 1 day) | | 479 (47.7) | | 0.2 | | 0.2, 0.3 | | 365 (75.1) | 0.5 | | 0.5, 0.5 | |
| Readmissions within 30 days | | 114 (11.3) | | 0.0 | | 0.0, 0.0 | | 101 (20.8) | 0.1 | | 0.1, 0.1 | |
| CC admissions^g^ | | 62 (6.2) | | 0.0 | | 0.0, 0.0 | | 55 (11.3) | 0.0 | | 0.0, 0.0 | |

^a^GP consultations included all consultations at a general practice, including those with a GP and those with a nurse. ^b^Drug codes for prescription cost analysis are presented in **Supplemental Table 5**. Biologics prescribed for the treatment of EGPA (mepolizumab, omalizumab, benralizumab, dupilumab, and rituximab) were not included, as these are predominantly prescribed and administered in the hospital setting and so are not well captured in the Clinical Practice Research Datalink Aurum database, which captures primary care data. ^c^Prescriptions for systemic corticosteroids commonly used in the treatment of EGPA (oral prednisone, oral prednisolone, oral methylprednisolone, and IV/IM methylprednisolone). ^d^Prescriptions for immunosuppressants commonly used in the treatment of EGPA (azathioprine, cyclophosphamide, mycophenolate mofetil, and methotrexate. ^e^Only attended outpatient appointments have been included. ^f^Admission with an admission date and a discharge date on the same day. ^g^CC included both ICU and HDU admissions.

A&E, accident & emergency; CC, critical care; CI, confidence interval; EGPA, eosinophilic granulomatosis with polyangiitis; GP, general practitioner; HCRU, healthcare resource utilisation; HDU, high-dependency unit; ICU, intensive care unit; IM, intramuscular; IV, intravenous; PY, person-years; SUA, severe uncontrolled asthma.

## Supplemental Table 4 HCRU annualised total costs in the incident EGPA cohort and matched general population and SUA cohorts (patients with ≥1 HCRU claim)

| Source of HCRU | Incident EGPA cohort,  median (IQR) | Incident EGPA cohort,  mean (SD) | Matched general population cohort,  median (IQR) | Matched general population cohort,  mean (SD) | Matched SUA cohort,  median (IQR) | Matched SUA cohort,  mean (SD) |
| --- | --- | --- | --- | --- | --- | --- |
| **Total** | **6,219 (3,272–12,153)** | **14,261 (43,916)** | **891 (256–2,518)** | **2,556 (8,654)** | **1,877 (835**–**4,129)** | **3,615 (6,598)** |
| **Outpatient care** |  |  |  |  |  |  |
| GP consultations^a^ | 319 (173–552) | 426 (441) | 152 (73–272) | 224 (299) | 241 (137–397) | 314 (290) |
| GP prescriptions  (All drugs of interest^b^) | 348 (198–620) | 484 (458) | 30 (6–124) | 138 (307) | 236 (94–500) | 370 (430) |
| Outpatient visits | 2,100 (1,159–3,412) | 2,544 (2,032) | 411 (157–929) | 778 (1,228) | 538 (238–1,143) | 930 (1,402) |
| **A&E visits** | 163 (86–392) | 361 (836) | 94 (52–197) | 188 (530) | 114 (62–232) | 226 (405) |
| **Hospital admissions** |  |  |  |  |  |  |
| Inpatient admissions (all) | 3,328 (1,398–6,686) | 11,365 (45,095) | 861 (305–2,114) | 2,557 (9,926) | 1,027 (347–2,714) | 2,543 (6,086) |
| **CC admissions^c^** | 588 (248–1,462) | 2,502 (8,194) | 347 (201–672) | 831 (2,193) | 323 (241–619) | 665 (1,034) |

^a^GP consultations included all consultations at a general practice, including those with a GP and those with a nurse. ^b^Drug codes for prescription cost analysis are presented in **Supplemental Table 5**. Biologics prescribed for the treatment of EGPA (mepolizumab, omalizumab, benralizumab, dupilumab, and rituximab) were not included, as these are predominantly prescribed and administered in the hospital setting and so are not well captured in the Clinical Practice Research Datalink Aurum database, which captures primary care data. ^c^CC included both ICU and HDU admissions.

A&E, accident & emergency; CC, critical care; EGPA, eosinophilic granulomatosis with polyangiitis; GP, General Practitioner; HCRU, healthcare resource utilisation; HDU, high-dependency unit; ICU, intensive care unit; IQR, interquartile range; SD, standard deviation; SUA, severe uncontrolled asthma.

## Supplemental Table 5 Drug codes for prescription cost analysis

| Drug class | ATC code | Morbidity |
| --- | --- | --- |
| Corticosteroids - topical GI | A07EA | GI inflammation |
| Mesalazine and similar agents | A07EC* | GI inflammation |
| Insulin | A10A* | Diabetes |
| Glucose lowering drugs | A10B* | Diabetes |
| Vitamin D & analogues | A11CC* | Osteoporosis, CKD |
| Calcium supplement | A12A* | Osteoporosis, CKD |
| Antithrombotics | B01* | Thrombosis |
| Antihemorrhagics | B02* | Haemoptysis |
| Cardiac glycosides | C01A* | Cardiomyopathy |
| Antiarrhythmics | C01B* | Cardiomyopathy |
| Vasodilators | C01D* | Cardiomyopathy |
| Ranolazine | C01EB18 | Ischaemia |
| Antihypertensives | C02* | Hypertension |
| Diuretics | C03* | Heart failure, CKD, cardiomyopathy, |
| Beta blockers | C07* | Cardiomyopathy, heart failure, CKD, ischaemia |
| Calcium channel blockers | C08* | Ischaemia, cardiomyopathy, CKD |
| ACE Inhibitors & Angiotensin-II receptor blockers | C09* | Hypertension, heart failure, cardiomyopathy, CKD |
| Statins, Fibrates and other lipid lowering agents | C10* | Dyslipidaemia |
| Cicatrizants | D03A* | Skin ulcers |
| Corticosteroids -topical | D07* | Inflammation |
| Raloxifene | G03XC01 | Osteoporosis |
| Vasopressins | H01BA* | Haemoptysis |
| Glucocorticoids -systemic | H02AB* | Inflammation |
| Antibacterials | J01* | Infections |
| Nintedanib | L01EX09 | Pulmonary fibrosis |
| Immunosuppressants | L04A* | Inflammation |
| Colchicine | M04AC01 | Pericarditis |
| Bisphosphonates | M05BA*, M05BB* | Osteoporosis |
| Antiepileptics | N03* | Seizures |
| Antipsychotics | N05A | Psychosis |
| Antiallergics - nasal | R01AC* | Asthma |
| Corticosteroids – nasal | R01AD* | Asthma |
| Adrenergics, corticosteroid, anticholinergics - inhalants | R03* | Asthma |
| Antihistamines - systemic | R06* | Allergy |
| Cyclophosphamide | L01AA01 | Inflammation |
| Rituximab | L01FA01 | Inflammation |
| Dupilumab | D11AH05 | Asthma |

*Denotes a wild card.

ACE, angiotensin-converting enzyme; ATC, Anatomical Therapeutic Chemical classification; GI, gastrointestinal; CKD; chronic kidney disease.

# Supplemental figures

## Supplemental Figure 1 Patient flow diagram

^a^GPs considered as duplicated by CPRD were excluded. ^b^The ID must have been within the CPRD-validated study period. ^c^Diagnoses for exclusion were Wegener's granulomatosis or granulomatosis with polyangiitis, microscopic polyangiitis, polyarteritis nodosa, Takayasu arteritis, and giant cell arteritis. ^d^Randomly selected patients matched by age, sex, and GP practice among patients that fulfilled the eligibility criteria for the matched general population or matched SUA cohorts.

A&E, accident and emergency; CPRD, Clinical Practice Research Datalink; EGPA, eosinophilic granulomatosis with polyangiitis; GP, general practitioner; HES, Hospital Episode Statistics; HES APC, HES Admitted Patient Care; ID, index date; ONS, Office for National Statistics; SUA, severe uncontrolled asthma.

## References

1.	Global Initiative for Asthma. Global strategy for asthma management and prevention, 2023. <https://ginasthma.org/2023-gina-main-report/>. Accessed 11 November 2025.

2.	Guillevin L, Pagnoux C, Seror R, Mahr A, Mouthon L, Toumelin PL, et al. The Five-Factor Score revisited: assessment of prognoses of systemic necrotizing vasculitides based on the French Vasculitis Study Group (FVSG) cohort. *Medicine (Baltimore).* 2011;90(1):19-27.
